# Reluctance to seek pediatric care during the COVID-19 pandemic and the risks of delayed diagnosis

**DOI:** 10.1186/s13052-020-00849-w

**Published:** 2020-06-29

**Authors:** Benedetta Ciacchini, Francesco Tonioli, Cinzia Marciano, Maria Grazia Faticato, Elena Borali, Alessio Pini Prato, Enrico Felici

**Affiliations:** 1 Department of Translational Medicine, Università degli Studi del Piemonte Orientale Amedeo Avogadro Facoltà di Medicina e Chirurgia, Novara, Piemonte Italy; 2 Azienda Ospedaliera Nazionale Santi Antonio e Biagio e Cesare Arrigo Alessandria, Pediatric and Pediatric Emergency Unit, Alessandria, Piemonte Italy; 3 Azienda Ospedaliera Nazionale Santi Antonio e Biagio e Cesare Arrigo Alessandria, Pediatric Surgery Unit, Alessandria, Piemonte Italy

**Keywords:** COVID-19, Delayed diagnosis, Pediatric emergency

## Abstract

Since the outbreak of COVID-19 pandemic, the number of cases registered worldwide has risen to over 3 million. While COVID-19 per se does not seem to represent a significant threat to the pediatric population, which generally presents a benign course and a low lethality, the current emergency might negatively affect the care of pediatric patients and overall children welfare. In particular, the fear of contracting COVID-19 may determine a delayed access to pediatric emergency facilities. Present report focuses on the experience of The Children Hospital in Alessandria (northern Italy). The authors document a drop in the number of admissions to the emergency department (A&E) during the lock-down. They will also focus on four emblematic cases of pediatric patients who were seen to our A&E in severe conditions. All these cases share a significant diagnostic delay caused by the parents’ reluctance to seek medical attention, seen as a potential risk factor for COVID-19 contagion. None was found positive to all COVID-19 swab or immunologic testing. All in all, our data strongly support the importance of promoting a direct and timely interaction between patients and medical staff, to prevent the fear of COVID-19 from causing more harm than the virus itself.

To the Editor,

Since the outbreak of Coronavirus Disease 2019 (COVID-19) in Wuhan (China), in the early December 2019, the spread of the infection has become pandemic, affecting at present over 100 countries [1].

As of April 29th there are currently 3,190,584 cases registered worldwide, of which 1,187,184 in Europe (37.2% of the cases worldwide) [2]. In Italy 203,591 cases of COVID-19 and 27,682 deaths have been reported so far. Only 1.8% of these cases occurred in pediatric age (0–18 years) [3].

The currently available literature, albeit still limited, indicates that children of all ages are susceptible to COVID-19 but with a more benign course [4]. In particular, a recent study conducted on more than 2000 pediatric cases described a low-to-moderate disease severity in 50.9 and 38.8% of patients, respectively, and a completely asymptomatic course in 4.4% [5]. Noteworthy, the overall reported lethality of COVID-19 in pediatric patients in Italy confirmed to be very low accounting for just about < 0.1% [3].

While COVID-19 per se does not seem to represent a significant threat to the pediatric population, the current emergency might cause indirect detrimental consequences in the management of pediatric patients and on overall children health.

It has been reported that the fear of contracting COVID-19 infection determines a delay of access to pediatric emergency facilities [6]. In the present report, the authors will support this observation by describing the experience at The Children Hospital, Alessandria, Italy.

The Cesare Arrigo Children’s Hospital is equipped with a third level emergency department (A&E), which admits around 21,000 patients per year. It is located in the city of Alessandria, in the north-west of Italy, very close to an infectious outbreak that currently counts a total of 3346 confirmed COVID-19 cases, (12.9% of total Piedmont’s cases, the second most Italian region involved in this pandemic outbreak) [7].

We compared the number of admissions to our pediatric A&E during March 2020 to the corresponding time frame of the previous year (Table 1). Our data demonstrated a 76% reduction of the number of admissions with a clear drop from 1934 to 461 during the same timespan (Fig. 1).

**Table 1:** Table summarising the data presented in the study

	March 2019		March 2020	
Total number of access to pediatric A&E	1934		461	
Red: critical patient, top priority	5	0,3%	2	0,4%
Yellow: subcritical patient, immediate priority	235	12%	58	13%
Green: low priority, deferrable care	1441	75%	378	82%
White: non urgent patient	253	13%	23	5%

**Fig. 1:**
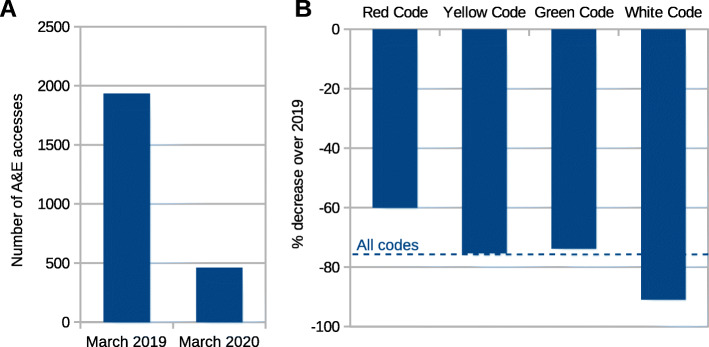
**a**, histogram showing a drop in the total number of pediatric A&E accesses during March 2019 and March 2020, respectively. **b**, percent decrease of A&E access number (relative to March 2019) by severity code. Horizontal dashed line shows the average drop (without taking into account the code)

Interestingly, we observed a significant reduction of the percentage of patients admitted for trivial issues (i.e. white codes and inappropriate A&E triages) and a mild decrease of that of patients who were admitted for serious conditions (i.e. green and yellow codes, respectively). Along this line, the number of critical cases (red codes) proved to be only slightly reduced when compared to the previous period (Fig. 1).

This phenomenon can be explained, at least in part, by the effectiveness of the restrictive measures of disease prevention and containment. In the pediatric population, infectious diseases represent amongst the most common issues that frequently require admission for medical treatment [8]. Therefore, the isolation of children away from places of aggregation and the consequent reduction of contacts has lowered the incidence of infectious diseases and their sequelae. Moreover, the lock-down has lowered the likelihood of experiencing traumas related to traffic, school, and sport injuries.

On the other hand, it has been shown that in the context of the current pandemic the population is discouraged from accessing hospitals even when urgent medical intervention would be mandatory, resulting in a greater severity of the patient’s conditions at the time of A&E admission. Similarly to what observed in the adult population, which showed a reduction of admissions to A&E for strokes [9] or cardiac diseases [10], reluctance in seeking medical attention has also been described in the pediatric context [6], and our Centre is no exception.

In the following section we report four cases of pediatric patients who arrived in March at our A&E in severe conditions, greatly worsened by the delayed access. Due to the fear of contracting COVID-19, these patients have been kept home longer than it would happen outside the sanitary emergency.

A 3 years old boy affected by Down Syndrome with a diagnosis of mild thrombocytopenia was admitted to emergency department showing generalized skin purpura with severe thrombocytopenia, that required immediate platelet transfusion. His parents had decided to skip the scheduled follow-up appointments because they were afraid of the COVID-19 infections. For the same reason, when the first symptoms showed up, they waited for over 1 week, even though they had undergone training for recognizing those symptoms requiring immediate medical care. He was eventually diagnosed with acute myeloid leukemia.

One child complained of neck pain with walking abnormalities and weight loss for almost 1 month. He was never seen by a doctor until he started with morning vomiting. When he arrived in Hospital he showed cerebellar signs, and a cerebellar mass with a diameter of 5 cm was found. The mass, pressing on the IV ventricle, caused a triventricular hypertensive hydrocephalous and compressed and dislocated the brain stem, threatening the life of the patient. The child was then admitted in ICU (Intensive Care Unit).

A 2 years old boy was kept at home for 1 week with high fever and a cough. After his pediatrician visited him during a video-call he was brought to the hospital. At arrival he showed severe respiratory distress and was immediately admitted to ICU. He was diagnosed with severe bacterial pneumonia with a massive parapneumonic pleural effusion requiring thoracic drainage and subsequent thoracoscopic decortication.

Finally, a child complained abdominal pain, high fever and vomiting for 5 days. At admission to A&E the child showed symptoms suggestive for acute appendicitis, which was supported by abdominal ultrasound and blood tests. The child underwent urgent surgery that allowed confirming an acute perforated appendicitis causing a stercoraceous peritonitis.

All those patients tested negative to COVID-19 swab test confirming their non-COVID-19 issues.

Overall, we observed that while the total number of pediatric admissions to the A&E has decreased, the type of issues leading the families to seek for urgent medical treatment has drastically changed: trivial issues as well as infections, post-infectious consequences and serious traumas have almost disappeared.

However, serious diseases still occur and many children, that would have been otherwise visited by a general practitioner (family paediatrician in Italy) or seen earlier to the A&E, showed a significant and dangerous diagnostic delay at admission. During the current pandemic, patients are more likely to be admitted to A&E in life-threatening conditions, often requiring immediate emergency surgery, transfusions or hospitalization in ICU. The cases herein described all share a clinical profile worsened by the diagnostic delay caused by the widespread tendency of parents to avoid hospitals and pediatricians’ assistance, that is seen as a potential risk factor for COVID-19 contagion.

These observations on one hand raise interesting questions regarding how appropriately the A&E service is used in non-pandemic times. On the other hand they underline the utmost importance of medical assessment as the parents on their own are not able to adequately discriminate the severity of a clinical condition.

In the current sanitary emergency, telemedicine may represent a valid alternative for a first evaluation of certain conditions. However, our experience underlines how this modality should be employed with extreme care, as the physical examination remains crucial for a correct and timely evaluation. Therefore, access to first-level healthcare in the territory should be ensured, enhanced and promoted, even for apparently benign symptoms, which may eventually suggest the urgent need for A&E advice or admission. At the same time, it is important to encourage parents to interact with medical staff when necessary, to prevent the fear of COVID-19 from causing more harm than the virus itself.

## Data Availability

All data generated or analysed during this study are included in this published article.
